# Hemispherotomy for Pediatric Post-Traumatic Epilepsy

**DOI:** 10.3390/brainsci16060657

**Published:** 2026-06-22

**Authors:** Habib E. Akouri, Samuel B. Tomlinson, Kevin Wojcik, Nankee K. Kumar, Kathleen Galligan, Sudha K. Kessler, Benjamin C. Kennedy

**Affiliations:** 1 Perelman School of Medicine, University of Pennsylvania, Philadelphia, PA 19104, USA; habib.akouri@pennmedicine.upenn.edu (H.E.A.);; 2 Department of Neurosurgery, University of Pennsylvania, Philadelphia, PA 19104, USA; samuel.tomlinson@pennmedicine.upenn.edu; 3 Department of Neurosurgery, Cooper University Hospital, Camden, NJ 08103, USA; 4 Division of Neurosurgery, Children’s Hospital of Philadelphia, 3401 Civic Center Boulevard, Philadelphia, PA 19104, USA; 5 Division of Neurology, Children’s Hospital of Philadelphia, 3401 Civic Center Boulevard, Philadelphia, PA 19104, USA

**Keywords:** hemispherotomy, traumatic brain injury, post-traumatic epilepsy, epilepsy, pediatrics

## Abstract

**Highlights:**

**What are the main findings?**

- Among five children with refractory post-traumatic epilepsy following traumatic brain injury (TBI), lateral trans-sylvian hemispherotomy achieved complete seizure freedom (Engel Class I) in all cases, with no surgical complications.
- Anti-seizure medication burden decreased from a median of five agents preoperatively to one postoperatively.

**What are the implications of the main findings?**

- Carefully selected children with lateralized post-traumatic epilepsy may achieve excellent seizure freedom and functional outcomes after hemispherotomy.
- Minor contralateral radiographic abnormalities should not automatically preclude hemispherotomy when electroclinical data consistently lateralize seizure onset.

**Abstract:**

**Objective:** Hemispherotomy is an effective treatment for select forms of drug-resistant hemispheric epilepsy, including perinatal stroke, Rasmussen’s encephalitis, and Sturge–Weber syndrome. Post-traumatic epilepsy (PTE) has been reported to occur in ~10% of children following traumatic brain injury (TBI). TBI has not been extensively evaluated as an indication for hemispherotomy, as its effects are rarely unilateral. Here, we report the results from five pediatric cases of hemispherotomy for drug-resistant hemispheric PTE. **Methods:** A retrospective review was performed of all pediatric patients with drug-resistant PTE secondary to TBI who underwent hemispherotomy between 2018 and 2022 at the Children’s Hospital of Philadelphia (*n* = 5). All patients initially underwent craniectomy and subsequent cranioplasty due to TBI; criteria for hemispherotomy were met in the following years, leading to a recommendation for hemispherotomy at the epilepsy surgery conference. Clinical characteristics, seizure and functional outcomes, and postoperative complications were reviewed. Seizure outcomes were classified according to the Engel criteria. **Results:** Five children (median age: 8.3 years, range: 5.0–10.3 years) with drug-resistant PTE underwent lateral trans-sylvian hemispherotomy. TBI etiology included non-accidental trauma (*n* = 3) and motor vehicle accidents (*n* = 2). All patients exhibited Engel Class Ia seizure outcomes (median follow-up: 15 months, range: 5–39 months), with a reduction in anti-seizure medications from a median of five preoperatively to one postoperatively. No patient experienced re-operation. Neuropsychological outcomes were patient-specific, with most exhibiting a mix of gains and challenges after surgery. **Conclusion:** We demonstrate the use of hemispherotomy to treat drug-resistant, hemispheric PTE in five children, with excellent reduction in seizure frequency and mixed or improved neuropsychological outcomes.

## 1. Introduction

Traumatic brain injury (TBI) is a leading cause of pediatric morbidity and mortality in the United States [1], accounting for over 800,000 pediatric emergency visits each year [2]. Post-traumatic epilepsy (PTE) is a serious neurological sequela of TBI. The incidence of PTE following TBI varies across the literature [3,4,5,6], with the largest pediatric studies finding that ~10% of children develop PTE following TBI [7]. PTE and subsequent treatment with anti-seizure medications (ASMs) can have deleterious social, behavioral, physical, and cognitive effects [3], and small pediatric series have reported progression to drug-resistant epilepsy [8].

The treatment of drug-resistant PTE is challenging. The pathologic underpinnings of PTE—including encephalomalacia, gliosis, and diffuse axonal injury—are commonly multifocal, and post-traumatic changes to the brain can complicate the localization of epileptogenic foci and surgical resection [9,10,11]. Neuromodulation treatments (e.g., vagus nerve stimulation) may reduce seizure burden in some patients, but these therapies are not curative and are generally understudied in pediatric drug-resistant PTE [11,12,13,14].

Hemispherotomy has been demonstrated as a highly effective treatment for select forms of drug-resistant hemispheric epilepsies such as hemispheric malformations, perinatal stroke, Sturge–Weber syndrome, and Rasmussen’s encephalitis [15,16]. Although TBI has not been rigorously evaluated as an indication for hemispherotomy, this approach may be considered when the traumatic insult and foci of seizure onset are primarily unilateral. Rare reports have documented the use of hemispherotomy for PTE, including a recent case series with three PTE cases, as well as a case of West syndrome after abusive head trauma [17,18].

Here, we report the outcomes of five children with severe, lateralized, drug-resistant PTE who underwent trans-sylvian hemispherotomy at a single pediatric epilepsy center. We describe selection criteria, perioperative course, seizure outcomes, and neurocognitive course.

## 2. Methods

### 2.1. Clinical Cohort

A retrospective, single-center study was performed from an IRB-approved epilepsy surgery outcomes registry. All cases from 2018 to 2022 were reviewed. Inclusion criteria were age younger than 18 years at the time of hemispheric surgery, history of TBI, drug-resistant epilepsy as defined by the International League Against Epilepsy (ILAE), and a minimum follow-up of five months after surgery. All patients were discussed at the multidisciplinary epilepsy surgery conference at the Children’s Hospital of Philadelphia to determine candidacy for hemispheric surgery. Patients with severe, widespread multi-lobar, primarily unilateral injury, with ipsilateral seizure onset as determined by semiology and electroencephalogram (EEG), were considered candidates for hemispherotomy. Surgical candidates required severe, predominantly unilateral hemispheric injury with concordant electrographic and semiologic lateralization. Contralateral radiographic abnormalities were not a contraindication to hemispherotomy, provided they were deemed by consensus review to be relatively minor and lacked any electroclinical evidence of independent epileptogenicity. MEG was used as an adjunct in two patients. Invasive monitoring was not performed in any patient.

### 2.2. Hemispherotomy

All surgeries were performed by a single surgeon (BCK). Patients underwent lateral, trans-sylvian peri-insular hemispherotomy via the technique described initially by Schramm et al. [19], with modifications described further by our group [20]. Post-operative magnetic resonance imaging (MRI) was obtained to verify the completeness of disconnection.

### 2.3. Data Collection

Demographic and preoperative clinical data were abstracted, including sex, age at TBI, age at surgery, mechanism of trauma, and prior surgical intervention for TBI. Epilepsy characterization included preoperative seizure frequency and semiology, ASMs, and preoperative neurologic condition. Preoperative MRI and scalp EEG were reviewed in all patients, and MEG in selected cases. Postoperative neuropsychological assessments were obtained in all patients. Standardized instruments—individualized for each patient—were utilized to assess intellectual functioning, academic achievement, domain-specific performance (language, attention, executive functioning, memory, visuospatial abilities, fine motor skills, adaptive functioning), and behavioral/emotional functioning. Instruments included WISC-V, WPPSI-IV, Woodcock–Johnson Tests of Cognitive Abilities and Achievement [Fourth Edition], WIAT-IV, CELF-5, NEPSY-II, ChAMP, VMI-6, D-KEFS, TEA-Ch, BASC-3, BRIEF-2, and ABAS-3, and were personalized per subject by the evaluating neuropsychologist. Cognitive outcome characterizations in Table 1 reflect overall clinical impressions documented by the evaluating neuropsychologist following assessment.

### 2.4. Clinical Outcomes

The primary measure of interest was seizure outcome defined by the Engel classification system (Class I: free of disabling seizures; II: rare disabling seizures; III: worthwhile improvement; IV: no worthwhile improvement). Secondary outcomes included the following: number of ASMs, surgical complications, reoperations, new postoperative deficits, and neuropsychological changes as noted through formal and/or subjective parental evaluation.

## 3. Results

### 3.1. Demographics

Five patients met the inclusion criteria (Table 1). Detailed TBI and epilepsy history for each patient can be found in Appendix A. All patients were male. Median age at the time of TBI was 22.2 months (mean: 22.5 months; range 1.3–64.0 months). TBI mechanism included non-accidental trauma (*n* = 3) and motor vehicle accident (*n* = 2). All patients underwent an index neurosurgical operation related to their TBI. Four patients underwent decompressive hemicraniectomy for management of elevated intracranial pressure and subsequent cranioplasty. One patient (Patient 3) underwent autologous cranioplasty for a growing skull fracture with brain herniation through a bony and dural defect. Patient 2 underwent ventriculoperitoneal shunt placement due to hydrocephalus, and Patient 1 underwent baclofen pump placement six years after his initial injury for TBI-related spasticity.

### 3.2. Epilepsy Characterization

**Figure 1:**
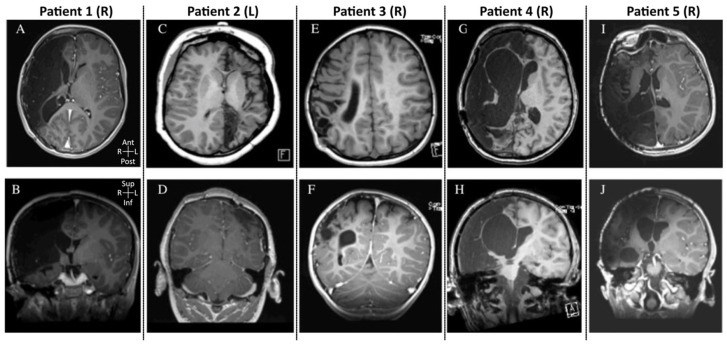
(**A**–**J**) Preoperative axial (top) and coronal (bottom) T1-weighted MRI images for patients 1–5, indicating the affected hemisphere (R: right, L: left). The figure itself labels each column as Patients 1–5, alongside the affected hemisphere labeled (and defined in the figure caption). Top and bottom images are defined as axial and coronal per the figure caption.

Median duration of epilepsy prior to surgery was 4.9 years (mean: 4.6 years; range 1.7–8.1 years). All patients had focal clinical seizures with motor correlate; patients 4 and 5 also experienced progression to bilateral tonic–clonic seizures. Patients 1 and 5 had subclinical seizures in addition to clinical seizures. The median number of ASMs trialed prior to surgery was five (mean: 4.8; range 3–7). Scalp EEG localized seizure onset in four patients; MEG aided localization in Patient 2 and corroborated findings in Patient 4. All seizures were lateralized to the hemisphere of initial injury as seen on MRI (Figure 1). Three of five patients—Patient 2 (Figure 1C,D), 4 (Figure 1G,H), and 5 (Figure 1I,J)—also exhibited injury of the contralateral hemisphere on preoperative imaging.

### 3.3. Seizure Outcomes

**Figure 2:**
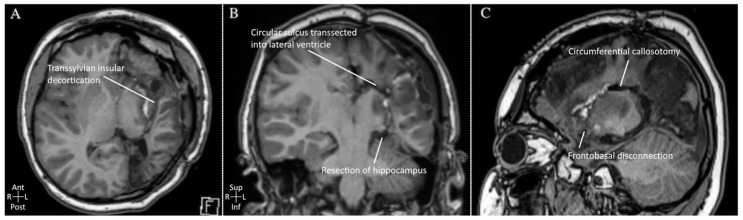
Axial (**A**), coronal (**B**), and sagittal (**C**) T1-weighted MRI images for a representative patient (Patient 2) demonstrating postoperative hemispheric disconnection following lateral trans-sylvian hemispherotomy. Key anatomic steps are highlighted (note that not all critical steps in the disconnection are illustrated). Patient 2 was selected because a single set of axial, coronal, and sagittal images most clearly captured the major steps of hemispheric disconnection.

After undergoing lateral, trans-sylvian hemispherotomy, postoperative MRI confirmed complete disconnection in every case. Figure 2 demonstrates complete anatomic disconnection as an example (Patient 2). The median time to last follow-up was 15 months (range: 5–39 months). All patients exhibited Engel Class Ia outcome at last follow-up, and the median number of post-operative ASMs decreased from five pre-operatively to one (range: 0–2). No patient required additional neurosurgical procedures.

### 3.4. Secondary Outcomes

One patient experienced immediate improvement of preoperative motor deficit. Two patients had an expected transient worsening of preexisting motor deficit, which completely resolved by the latest follow-up. Two patients experienced post-operative complications. One had medication-related pancreatitis with pleural effusion requiring reintubation and a chest tube. One patient developed a delayed ipsilateral basal ganglia hemorrhage with self-limited headache, vomiting, and no new neurological deficit. Both patients recovered fully. No reoperations or post-hemispherotomy shunts were required.

### 3.5. Cognitive Outcomes

All five patients underwent formal postoperative neuropsychological testing, although timing varied widely (Table 1). Outcomes were patient-specific and generally mixed. Three patients (1, 4, 5) had an overall positive neuropsychological outcome when assessing both formal testing and parental reports. Two patients (2, 3) exhibited more mixed outcomes with specific areas of improvement and challenges noted postoperatively.

## 4. Discussion

Here, we report five children with drug-resistant PTE following TBI who successfully underwent lateral approach hemispherotomy resulting in Engel Class Ia seizure outcomes at a median follow-up of 15 months. ASMs were reduced from a median of five to one; three patients demonstrated neuropsychological or functional improvement, and one patient experienced near-immediate motor improvement from his preoperative baseline. No patients experienced major post-surgical complications or required additional neurological surgery.

There are limited reports of hemispherotomy for PTE in the neurosurgical literature. Several authors have suggested that TBI may be a potential indication for hemispheric surgery, but acknowledge that the decision to pursue surgery in such cases is nuanced, given the rarity of well-lateralized PTE following TBI. Examples in the literature of PTE treated successfully with hemispherotomy are sparse. In one report, an infant with a unilateral growing skull fracture and PTE exhibited motor recovery and seizure control after hemispherotomy [21]. Another report described a 25-year-old with PTE after a vehicular TBI who experienced seizure freedom and behavioral improvement after hemispherotomy [22]. Some hemispherotomy case series have included subsets of patients with PTE as their surgical indication. For example, one study of 12 hemispherotomy patients included a child with unilateral PTE who achieved Engel class Ic seizure control; they had one generalized seizure postoperatively at 5 months, thought to be secondary to a febrile illness, and remained seizure-free afterwards [23]. Various other series include hemispherotomy patients with PTE as a subset of their larger samples, but limited details are shared for these patients [24]. 

Together, these data support the idea that hemispherotomy can be a safe and effective treatment for PTE when seizure onset is determined to be well-lateralized. Our series aligns with and extends this literature by reporting uniform Engel Ia outcomes in five consecutively treated children, identified with careful presurgical evaluation and group consensus. Additionally, minor contralateral radiographic abnormalities—which were present in three of our five cases—did not preclude a favorable outcome.

The best rates of control of drug-resistant PTE are in patients who have identifiable seizure foci on EEG [25]. For instance, surgical resection for temporal lobe epilepsy associated with mesial temporal sclerosis (MTS) has been shown to achieve seizure freedom in approximately 60–70% of patients in randomized and meta-analytic data [26,27], and in PTE-specific cohorts, up to 77% of patients with MTS-related PTE achieve Engel Class I outcomes, with over 90% achieving Engel Class I or II [28]. However, in several patients with PTE, the localization of epileptogenic foci and their surgical resection is often complicated by multifocal post-traumatic changes such as encephalomalacia and gliosis [9,11]. Similarly, the diffuse cerebral injury caused by TBI often generates multiple epileptogenic foci, making surgical resection less likely to result in seizure freedom [10]. 

When surgery is not possible, neurostimulation may improve PTE [11]. Studies evaluating VNS in drug-resistant PTE in adults have shown successful reductions in seizure frequency [12,13,14]. In a large case–control study of patients in the VNS Therapy Patient Outcome Registry, Englot et al. found that seizure frequency decreased by 50% after three months and 73% after 24 months in patients with PTE [12]. The clinical response of PTE patients exceeded that of the matched non-PTE patients, who showed a 46% decrease and 57% decrease at three and 24 months, respectively. These results are in line with single-institution, retrospective studies that demonstrated an average 30–68% reduction in seizure frequency after VNS treatment [13,14]. Little data exist regarding the use of deep brain stimulation and responsive neurostimulation on drug-resistant PTE [29,30,31]. In the pediatric population, VNS has shown promising results in the treatment of non-traumatic drug-resistant epilepsy in children; however, there is little data on the efficacy in drug-resistant PTE [32,33].

Considering the severity of our patients’ epilepsy, widespread hemispheric injury, pre-existing deficits, concordant semiological and electrographic findings, and the hemispheric EEG localization of seizure onset, hemispherotomy was performed in lieu of a focal resection or neurostimulation. TBI has been considered a challenging indication for surgery. The resulting encephalomalacia and diffuse cerebral injury frequently produce multifocal epileptogenic networks across both hemispheres, which complicates surgical efficacy and planning and potential success. The present cohort differs from typical PTE populations in that all patients sustained severe and predominantly unilateral injury at a young age, demonstrated consistent multimodal lateralization, and an injury of sufficient severity to preclude focal resection after multidisciplinary consensus. Our cases highlight how appropriate patient selection, based on pre-operative imaging and epilepsy characterization, is crucial to the success of hemispherotomy in drug-resistant PTE. Although three patients demonstrated post-traumatic injury of the contralateral hemisphere on pre-operative imaging, semiology and EEG findings led the multidisciplinary epilepsy surgery conference to conclude that these more minor contralateral injuries would not preclude a favorable seizure outcome. Our results support these conclusions: well-selected patients with hemispheric PTE may have marked reduction or complete resolution of seizures after hemispherotomy. Furthermore, they may experience improvements in cognitive and motor function. 

The limitations of this study include its retrospective nature, small sample size, and limited follow-up duration. A longer follow-up will be important given the well-understood possibility of late recurrence and/or new contralateral seizures after hemispherotomy. Importantly, our results do not suggest that hemispherotomy is appropriate or effective for all patients with PTE, but only those with carefully determined criteria. Future work should include multicenter registries, utilizing standardized functional and cognitive assessments, and longer follow-ups, to clarify the efficacy and developmental impact of hemispherotomy for traumatic etiologies. An additional concern of this study is selection bias, as these patients were referred to a quaternary epilepsy center for surgical consideration. Thus, this selected cohort is more likely to have a suspicion of lateralized epilepsy than a general TBI population. Also, all procedures were performed by a single experienced surgeon at one institution. While this ensures technical consistency, it limits the generalizability of operative outcomes to other surgeons and centers. While no patients were lost to follow-up, child-protection proceedings due to the nature of non-accidental trauma may have impacted individual data completeness. 

## 5. Conclusions

Among five carefully selected children with drug-resistant PTE following TBI, hemispherotomy resulted in seizure freedom and low morbidity. Hemispherotomy should be considered when treating children with severe, well-lateralized, refractory PTE associated with injuries affecting predominantly one hemisphere.

## Figures and Tables

**Table 1:** **Cohort demographics, injury characteristics, epilepsy characterization, surgical outcomes, and cognitive testing.** Abbreviations: ADHD = attention-deficit/hyperactivity disorder; ASM = anti-seizure medication; EEG = electroencephalography; ICA = internal carotid artery; L = left; MEG = magnetoencephalography; MRI = magnetic resonance imaging; MVA = motor vehicle accident; NAT = non-accidental trauma; PTE = post-traumatic epilepsy; R = right; TBI = traumatic brain injury; VP = ventriculoperitoneal.

	Patient 1	Patient 2	Patient 3	Patient 4	Patient 5
DEMOGRAPHICS & TBI HISTORY					
**Sex**	M	M	M	M	M
**Lateralization**	R	L	R	R	R
**TBI mechanism**	MVA	NAT	NAT	MVA	NAT
**Age at TBI (months)**	23.2	2.0	1.3	64.0	22.2
**Prior surgical intervention(s)**	Hemicraniectomy + cranioplasty; Baclofen pump (6 years post-injury)	Hemicraniectomy + cranioplasty; VP shunt	Autologous cranioplasty (growing skull fracture)	Hemicraniectomy + cranioplasty	Hemicraniectomy + cranioplasty
**EPILEPSY CHARACTERIZATION**					
**Age at surgery (years)**	10.3	8.3	5.0	9.8	6.7
**Epilepsy duration pre-op (years)**	6.5	8.1	4.9	1.7	1.8
**Preoperative seizure frequency (per month)**	4–8 (1–2/week) at onset, escalated in 6 months leading up to surgery	>1/month, then weekly, up to several per day prior to surgery	Monthly, increasing in frequency approaching surgery	Multiple per week	2–3/day
**Seizure semiology**	Focal motor; subclinical; focal-to-bilateral tonic–clonic	Focal motor	Focal motor	Focal motor; bilateral tonic–clonic	Focal motor; bilateral tonic–clonic; subclinical
**ASMs trialed (n)**	7	5	6	3	3
**EEG/MEG findings**	Scalp EEG: ipsilateral	MEG: ipsilateral	Scalp EEG: ipsilateral	EEG + MEG: ipsilateral	Scalp EEG: ipsilateral
**Ipsilateral MRI pathology**	Remote right ICA-territory infarction with extensive cystic encephalomalacia and gliosis	Remote left hemispheric traumatic/ischemic injury with hemispheric atrophy, gliosis, and encephalomalacia	Chronic right fronttemporoparietal encephalomalacia following cranioplasty	Extensive right cerebral and cerebellar encephalomalacia with gliosis	Severe right hemispheric encephalomalacia
**Contralateral MRI abnormality (Y/N)**	No	Yes	No	Yes	Yes
**Contralateral abnormality description**	—	Focal right anterior medial cerebellar encephalomalacia and volume loss	—	Multifocal contralateral gliosis	Left frontal gliosis
**SURGICAL OUTCOMES**					
**Engel class at follow-up (months)**	1A (18 mo)	1A (15 mo)	1A (12 mo)	1A (39 mo)	1A (5 mo)
**Post-op ASMs at last follow-up**	2	0	1	0	1
**Complications**	none	none	none	Pancreatitis, chest tube, L peripancreatic fluid collection	Delayed ipsilateral basal ganglia hemorrhage with intraventricular hemorrhage
**COGNITIVE OUTCOMES**					
**Formal neuropsychological testing post-op (Y/N)**	Yes	Yes	Yes	Yes	Yes
**Timing of post-op assessment (months post-op)**	7.4	60.1	1.0	54.3	50.9
**Cognitive outcome**	**Strengths**: Energy, ability to participate in learning and cognitive activities, mood/anxiety, happiness **Weaknesses:** Distractibility **Global impression:** Improved	**Strengths**: General intellectual functioning, working memory, visual-spatial skills, and fine motor abilities **Weaknesses**: Verbal memory, confrontation naming, and adaptive functioning **Global impression:** Mixed	**Strengths:** Shown some nice gains over the course of his hospitalization and will continue to make gains in the upcoming months to years **Weaknesses**: Inattention/distractibility, executive functioning skills, processing speed, expressive language, pre-academic skills **Global impression:** Mixed	**Strengths:** Linguistic skills, comprehension, phonological processing, emotional and social skills, increasing independence **Weaknesses:** Fluid reasoning, processing speed **Global impression:** Improved	**Strengths:** Enhanced reasoning abilities, verbal comprehension, visual-spatial reasoning, fluid reasoning, visual discrimination, adaptive functioning, social-emotional functioning, and verbal memory **Global impression:** Improved

## Data Availability

No new data were created or analyzed in this study. Data sharing is not applicable to this article due to the retrospective nature of clinical records held under an established IRB registry.

## Abbreviations

ASM	Anti-seizure medication
MRI	Magnetic resonance imaging
PTE	Post-traumatic epilepsy
TBI	Traumatic brain injury

## Appendix A

Supplementary patient histories are provided for each subject included in the series.

**Patient 1** is a ten-year-old male who was involved in an all-terrain vehicle accident at the age of 23 months and suffered a traumatic brain injury (TBI) complicated by an internal carotid artery stroke due to a carotid dissection. He underwent a decompressive hemicraniectomy (DHC) at an outside institution. He developed subclinical seizures in the perioperative setting, detected on electroencephalogram (EEG) monitoring, which later resolved. He then began having unprovoked seizures at the age of four years, which were characterized by behavioral arrest with head version leftward and unresponsiveness lasting about 30 s, followed by post-ictal fatigue. He underwent placement of a baclofen pump for spasticity resulting from his TBI at age eight years. At the age of ten, the patient was admitted to our institution due to worsening seizure frequency. EEG monitoring recorded subclinical and subtle clinical seizures that localized to the area of his previous TBI. He was trialed on seven anti-seizure medications (ASMs) and was determined to have drug-resistant epilepsy. Magnetic resonance imaging (MRI) confirmed right hemispheric insult. A magnetoencephalography (MEG) study was foregone as it would have required shutting off his baclofen pump and was deemed an unnecessary risk given the corroboratory diagnostic testing.

**Patient 2** is an eight-year-old male who suffered non-accidental trauma (NAT) at three months old, requiring DHC and subsequent cranioplasty. He also underwent placement of a ventriculoperitoneal shunt for hydrocephalus. He developed epilepsy at three years of age, characterized by right-hand stiffening/jerking and associated paresthesias. He experienced behavior and learning issues as a result of his seizures and did not tolerate changes to his ASMs. He was trialed on five ASMs and was determined to have medically refractory epilepsy. The patient’s seizures were primarily clinical without a corresponding EEG correlate. MRI brain noted an asymmetrically small left cerebral hemisphere with gliosis and encephalomalacia, as well as some volume loss and encephalomalacia in the contralateral anterior medial right cerebellar hemisphere. MEG noted a mix of sharp and spike activity superimposed on a background of slowing in the left medial occipital lobe in an area surrounded by cystic encephalomalacia.

**Patient 3** is a four-year-old male who suffered NAT and TBI at five weeks of age with associated subdural hematoma and subarachnoid hemorrhage complicated by refractory subclinical status epilepticus. He underwent craniotomy and cranioplasty in the setting of a growing skull fracture. The patient went on to develop infantile spasms with hemihypsarrthymia which resolved. He subsequently developed focal epilepsy that remained refractory to six ASMs. His semiology included left arm clonic movements and abnormal eye movement. He had a mild left hemiparesis at baseline and a significant speech and language delay. MRI noted findings of chronic encephalomalacia changes in the right frontotemporoparietal region. EEG showed a clinical correlate with focal slowing and epileptiform discharges in the corresponding area of injury.

**Patient 4** is a nine-year-old male who suffered a severe TBI at the age of four years during a motor vehicle collision. He underwent a DHC due to malignant cerebral edema with resection of necrotic brain. His TBI was complicated by right- and left-sided cerebral infarctions. He subsequently developed chronic static encephalopathy manifested by left hemiparesis and learning difficulties. Semiology included left arm and leg stiffening and extension, occasionally progressing to generalized shaking. The patient was trialed on three ASMs, which decreased the frequency of his seizures, but he continued to experience multiple seizures daily. He underwent EEG and MRI/MEG, which were consistent with ictal onset in the residual right parasagittal frontal lobe.

**Patient 5** is a six-year-old male who presented with a seizure and was found to have a large right-sided subdural hematoma in the setting of NAT at 22 months of age. He underwent a DHC with subsequent titanium mesh cranioplasty. His hospital course was complicated by dysautonomia, which eventually resolved. He began having seizures at age five years, and his semiology was characterized by arrest of behavior with eyes and head turned to the left with bilateral hand automatisms. Despite trying three ASMs, the patient continued to experience two to four seizures throughout the day, with some generalizing to generalized tonic–clonic seizures. EEG noted diffuse voltage attenuation with sharp activity in the right temporal region and right central region, subclinical seizure onset. MRI notes severe encephalomalacia in the right cerebral hemisphere and gliosis in the left frontal lobe. MEG was nondiagnostic due to artifact from the titanium mesh cranioplasty.
